# Supplementary material for: Brain-wide organization of intrinsic timescales at single-neuron resolution
Source: bioRxiv. 2025 Aug 30:2025.08.30.673281. Preprint. [Version 1] doi: 10.1101/2025.08.30.673281 (PMC12407892; doi:10.1101/2025.08.30.673281)
Supplement: Supplement 1 [file media-1.pdf]

Supplementary Materials for:
Brain-wide organization of intrinsic timescales at single-neuron resolution

Yan-Liang Shi[1,*], Roxana Zeraati[2,3,*], International Brain Laboratory[4], Anna Levina[2,3,5,†] and Tatiana A. Engel[1,†]

[1]Princeton Neuroscience Institute, Princeton University, Princeton, NJ, USA
[2]Max Planck Institute for Biological Cybernetics, Tübingen, Germany
[3]University of Tübingen, Tübingen, Germany
[4]International Brain Laboratory, Virtual Entity
[5]Bernstein Center for Computational Neuroscience Tübingen, Tübingen, Germany
[*] These authors contributed equally to this work.
[†]Corresponding author e-mail: tatiana.engel@princeton.edu, anna.levina@uni-tuebingen.de

Contents

1 Supplementary Tables **2**

2 Supplementary Figures **7**

References

[1] Q. Wang, *et al.*, *Cell* **181**, 936 (2020).

[2] M. Song, *et al.*, *Proceedings of the National Academy of Sciences* **121**, e2415695121 (2024).

1 Supplementary Tables

Cortical region ID	Acronym	Region name
39	ACAd	Anterior cingulate area dorsal part
44	ILA	Infralimbic area
48	ACAv	Anterior cingulate area ventral part
104	AId	Agranular insular area dorsal part
111	AIp	Agranular insular area posterior part
119	AIv	Agranular insular area ventral part
184	FRP	Frontal pole cerebral cortex
329	SSp-bfd	Primary somatosensory area barrel field
337	SSp-ll	Primary somatosensory area lower limb
345	SSp-m	Primary somatosensory area mouth
353	SSp-n	Primary somatosensory area nose
361	SSp-tr	Primary somatosensory area trunk
369	SSp-ul	Primary somatosensory area upper limb
378	SSs	Supplemental somatosensory area
385	VISp	Primary visual area
394	VISam	Anteromedial visual area
409	VISl	Lateral visual area
417	VISrl	Rostrolateral visual area
425	VISpl	Posterolateral visual area
533	VISpm	Posteromedial visual area
541	TEa	Temporal association areas
723	ORBl	Orbital area lateral part
731	ORBm	Orbital area medial part
746	ORBvl	Orbital area ventrolateral part
879	RSPd	Retrosplenial area dorsal part
886	RSPv	Retrosplenial area ventral part
894	RSPagl	Retrosplenial area lateral agranular part
972	PL	Prelimbic area
985	MOp	Primary motor area
993	MOs	Secondary motor area
1002	AUDp	Primary auditory area
1011	AUDd	Dorsal auditory area
1027	AUDpo	Posterior auditory area
312782546	VISa	Anterior area
312782574	VISli	Laterointermediate area
312782628	VISpor	Postrhinal area

Supplementary Table 1. Cortical regions included in the analysis of the relationship between timescales and anatomical hierarchy scores. For each region, the table lists the brain region ID based on the Allen Common Coordinate Framework, its acronym, and full name [1].

Thalamic region ID	Acronym	Region name
127	AM	Anteromedial nucleus
149	PVT	Paraventricular nucleus of the thalamus
155	LD	Lateral dorsal nucleus of thalamus
170	LGd	Dorsal part of the lateral geniculate complex
181	RE	Nucleus of reuniens
218	LP	Lateral posterior nucleus of the thalamus
255	AV	Anteroventral nucleus of thalamus
362	MD	Mediodorsal nucleus of thalamus
366	SMT	Submedial nucleus of the thalamus
475	MG	Medial geniculate complex
575	CL	Central lateral nucleus of the thalamus
599	CM	Central medial nucleus of the thalamus
629	VAL	Ventral anterior-lateral complex of the thalamus
685	VM	Ventral medial nucleus of the thalamus
718	VPL	Ventral posterolateral nucleus of the thalamus
733	VPM	Ventral posteromedial nucleus of the thalamus
907	PCN	Paracentral nucleus
930	PF	Parafascicular nucleus
1020	PO	Posterior complex of the thalamus
1029	POL	Posterior limiting nucleus of the thalamus
1113	IAD	Interanterodorsal nucleus of the thalamus
560581563	PIL	Posterior intralaminar thalamic nucleus

Supplementary Table 2. Thalamic regions included in the analysis of the relationship between timescales and anatomical hierarchy scores. For each region, the table lists the brain region ID based on the Allen Common Coordinate Framework, its acronym, and full name [1].

Brain structure	Acronym	N	N_θ	Cutoff θ (s)	KS distance	p-value
All brain regions		10,279	1,641	1.2	0.023	0.12
Isocortex	CTX	2,439	553	0.7	0.026	0.29
Olfactory areas	OLF	468	158	0.2	0.035	0.71
Hippocampal formation	HPF	1,327	370	0.1	0.027	0.48
Cortical subplate	CTXsp	175	114	0.02	0.043	0.76
Striatum	STR	1,143	154	0.5	0.053	0.19
Pallidum	PAL	164	42	0.6	0.085	0.22
Thalamus	TH	2,601	463	0.4	0.024	0.50
Hypothalamus	HY	82	34	0.6	0.094	0.46
Midbrain	MB	914	286	1.1	0.027	0.66
Pons	P	215	93	1.2	0.048	0.76
Medulla	MY	446	246	0.6	0.045	0.14
Cerebellum	CB	305	143	0.8	0.046	0.39

Supplementary Table 3. Bootstrap-based statistical test to assess whether the timescale distribution is consistent with a power law. Results of the statistical test that estimates the p-value for rejecting the hypothesis that the timescales follow a power-law distribution in each major brain structure and in the data pooled from all regions. For each dataset, the table lists the number of neurons N, the number of neurons with timescales exceeding the optimal cutoff value N_θ, the optimal cutoff value θ, the KS distance to the best-fitting power law, and a p-value for rejecting the power-law hypothesis. A p-value greater than 0.1 implies that the power-law hypothesis cannot be ruled out. In all brain structures and in the data pooled from all regions, the p-values exceeded the 0.1 threshold, indicating that the timescales were consistent with a power-law distribution.

Brain structure	Acronym	N	N_θ	Cutoff θ (s)	KS distance	p-value
All brain regions		26,888	4,461	3.95	0.013	0.2201
Isocortex	CTX	6,489	770	5.47	0.024	0.5284
Olfactory areas	OLF	1,111	309	1.85	0.036	0.5702
Hippocampal formation	HPF	2,943	523	3.10	0.020	0.9377
Cortical subplate	CTXsp	498	104	2.86	0.073	0.3083
Striatum	STR	2,820	509	2.48	0.013	0.9998
Pallidum	PAL	357	64	3.57	0.070	0.6702
Thalamus	TH	4,794	771	3.67	0.019	0.8305
Hypothalamus	HY	270	105	2.24	0.049	0.8174
Midbrain	MB	2,043	568	3.13	0.025	0.6933
Pons	P	524	220	2.65	0.051	0.3198
Medulla	MY	787	253	2.37	0.044	0.4222
Cerebellum	CB	1,381	358	3.59	0.038	0.4001

Supplementary Table 4. Bootstrap-based statistical test to assess whether timescales estimated by single-exponential fits are consistent with a power-law distribution. Results of the statistical test that estimates the p-value for rejecting the hypothesis that the timescales follow a power-law distribution in each major brain structure and in the data pooled from all regions. For each dataset, the table lists the number of neurons N, the number of neurons with timescales exceeding the optimal cutoff value N_θ, the optimal cutoff value θ, the KS distance to the best-fitting power law, and a p-value for rejecting the power-law hypothesis. To compute the p-value, we generated an ensemble of $n = 100,000$ synthetic datasets. A p-value greater than 0.1 implies that the power-law hypothesis cannot be ruled out. In all brain structures and in the data pooled from all regions, the p-values exceeded the 0.1 threshold, indicating that the timescales were consistent with a power-law distribution. All neurons were included in this analysis.

Brain structure	Acronym	N	N_θ	R (lognormal)	p(lognormal)	R (exp)	p(exp)
All brain regions		10,279	1,641	0.031	0.54	1647.06	$< 1.0 \times 10^{-10}$
Isocortex	CTX	2,439	553	0.002	0.98	649.85	$< 1.0 \times 10^{-10}$
Olfactory areas	OLF	468	158	0.002	0.88	189.18	5.7×10^{-10}
Hippocampal formation	HPF	1,327	370	-0.68	0.46	481.78	$< 1.0 \times 10^{-10}$
Cortical subplate	CTXsp	175	114	-0.085	0.81	171.79	7.1×10^{-3}
Striatum	STR	1,143	154	0.002	0.95	312.02	$< 1.0 \times 10^{-10}$
Pallidum	PAL	164	42	-0.44	0.55	10.62	1.5×10^{-1}
Thalamus	TH	2,601	463	-0.72	0.37	710.41	$< 1.0 \times 10^{-10}$
Hypothalamus	HY	82	34	-0.046	0.37	25.44	9.0×10^{-3}
Midbrain	MB	914	286	-0.47	0.48	209.82	$< 1.0 \times 10^{-10}$
Pons	P	215	93	-0.20	0.63	52.71	2.3×10^{-7}
Medulla	MY	446	246	-1.86	0.14	164.08	$< 1.0 \times 10^{-10}$
Cerebellum	CB	305	143	-1.36	0.23	75.14	3.7×10^{-8}

Supplementary Table 5. Comparison of power-law fits with exponential and log-normal alternatives. Results of comparing the goodness of fit of the power-law model against the exponential and log-normal alternatives for the timescale distributions in each major brain structure and in the data pooled from all regions. For each dataset, the table lists the number of neurons N, the number of neurons with timescales exceeding the optimal cutoff value N_θ, the normalized log-likelihood ratio (LLR) between the power-law and the lognormal model R(lognormal) and the corresponding p-value p(lognormal), and the normalized LLR between the power-law and exponential model R(exp) and the corresponding p-value p(exp). A positive LLR indicates that the power-law model provides a better fit than the alternative, whereas a negative LLR favors the alternative.

2 Supplementary Figures

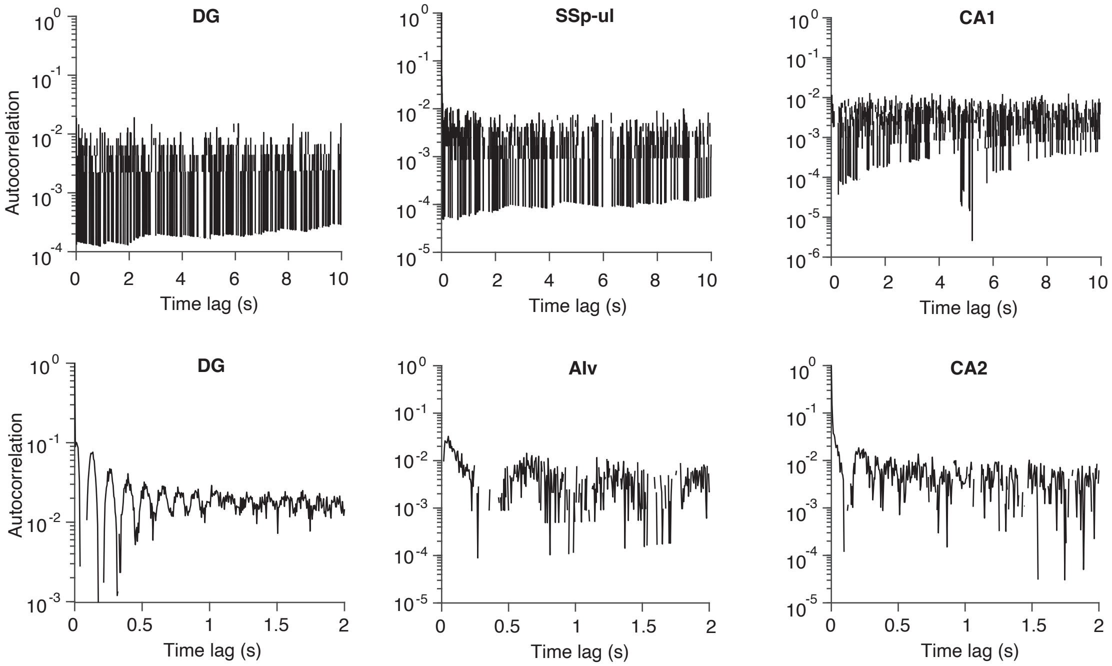

Supplementary Figure 1. Example autocorrelations that cannot be adequately captured by exponential decay functions. Autocorrelations of example single neurons poorly fitted by the exponential decay model (coefficient of determination $R^2 \leqslant 0.5$). The autocorrelation shapes were either dominated by noise (top row; DG: Dentate gyrus; SSpul: Primary somatosensory area upper limb; CA1: Field CA1) or oscillatory features (lower row; DG: Dentate gyrus; Alv: alveus; CA2: Field CA2).

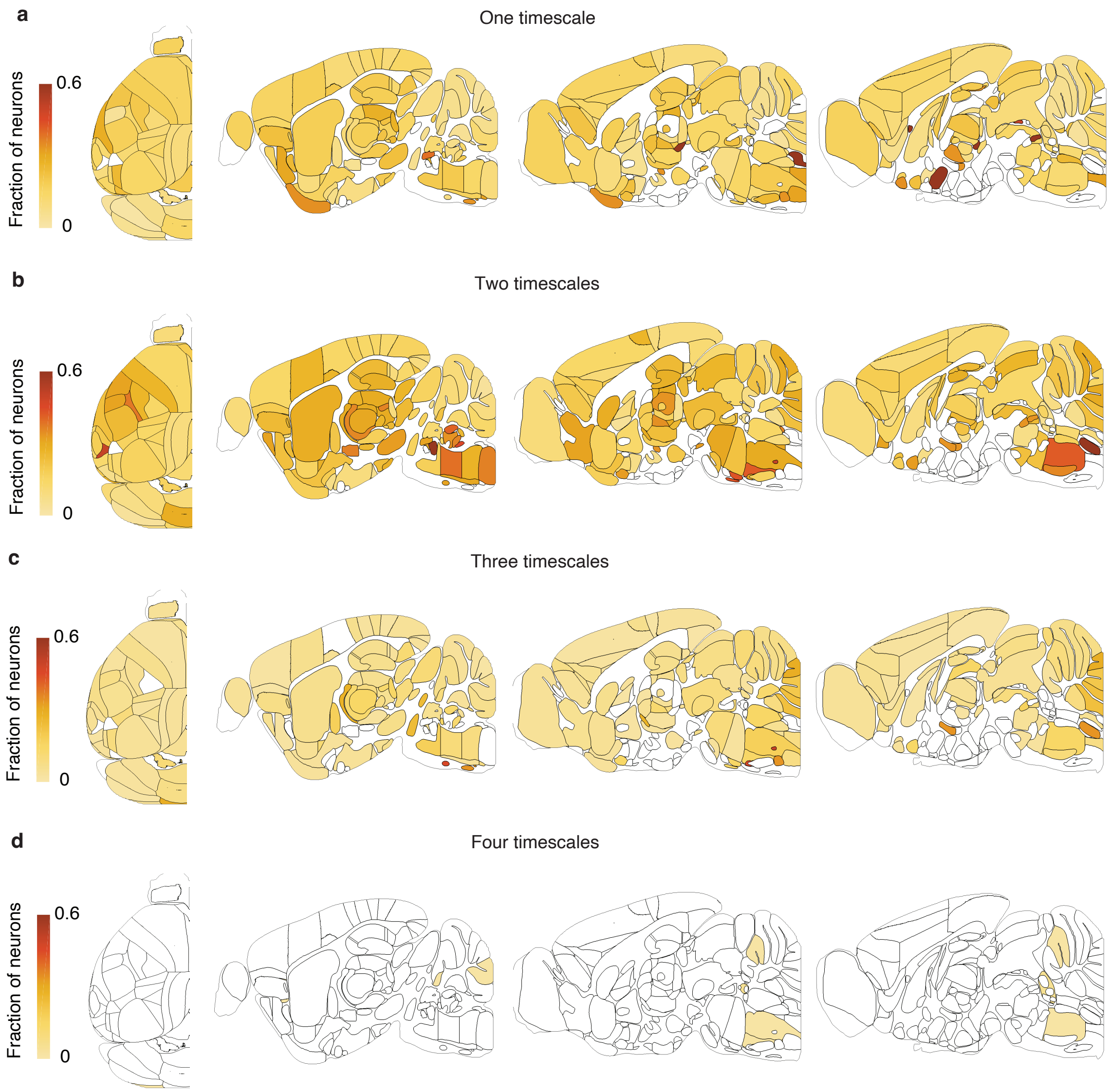

Supplementary Figure 2. Distribution of neurons with different number of timescales across the brain. **a**, Brain-slice plots (top and sagittal views) showing the fraction of neurons best fit by a single-timescale model in each brain region. **b**, Same as panel a for neurons best fit by a two-timescale model. **b**, Same as panel a for neurons best fit by a three-timescale model. **b**, Same as panel a for neurons best fit by a four-timescale model.

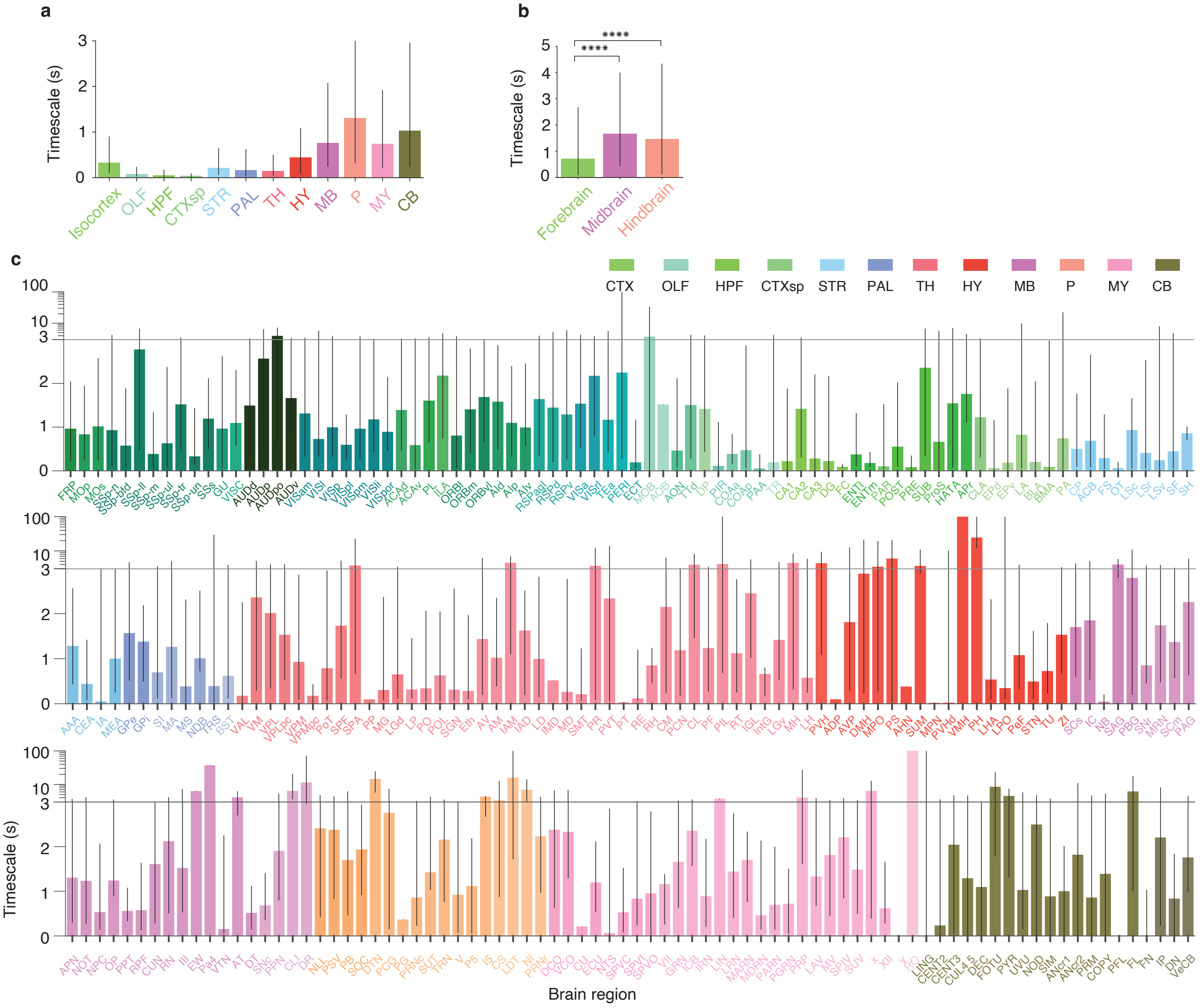

Supplementary Figure 3. A brain-wide map of intrinsic timescales estimated from single-exponential fit. a, Timescales in 12 major brain structures. Bar height indicates the median, and error bars represent the 25th to 75th percentiles. **b**, Timescales for the three major brain divisions: forebrain (including isocortex, olfactory areas, hippocampal formation, cortical subplate, striatum, pallidum, thalamus, and hypothalamus), midbrain, and hindbrain (including medulla, pons, and cerebellum). Bar height indicates the median (forebrain 0.72 s, $n = 19,979$ neurons; midbrain 01.66 s, $n = 2,144$; hindbrain 1.47 s, $n = 2,831$), and error bars represent the 25th to 75th percentiles. Timescales were significantly longer in the midbrain and hindbrain than in the forebrain (two-sided Wilcoxon rank sum test, $^{****}p < 10^{-5}$). **c**, Timescales for all brain regions across the 12 major brain structures indicated by color (CTX, isocortex; OLF, olfactory areas; HPF, hippocampal formation; CTXsp, cortical subplate; STR, striatum; PAL, pallidum; TH, thalamus; HY, hypothalamus; MB, midbrain; P, pons; MY, medulla; CB, cerebellum). Bar height indicates the median, and error bars represent the 25th to 75th percentiles of the timescale distribution. The y-axis transitions from a linear to logarithmic scale (horizontal line) to accommodate the wide distribution of timescales. All neurons were included in these analyses.

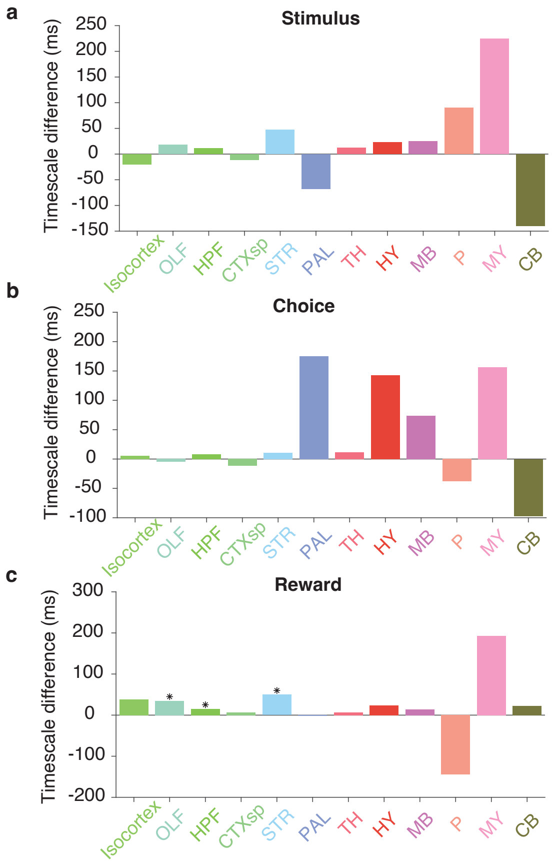

Supplementary Figure 4. Differences in timescales between selective and non-selective neurons for each task variable. **a**, Difference in median effective timescales between selective and non-selective neurons for the visual stimulus in each major brain structure. **b**, Same as panel a for choice. **c**, Same as panel a for reward.

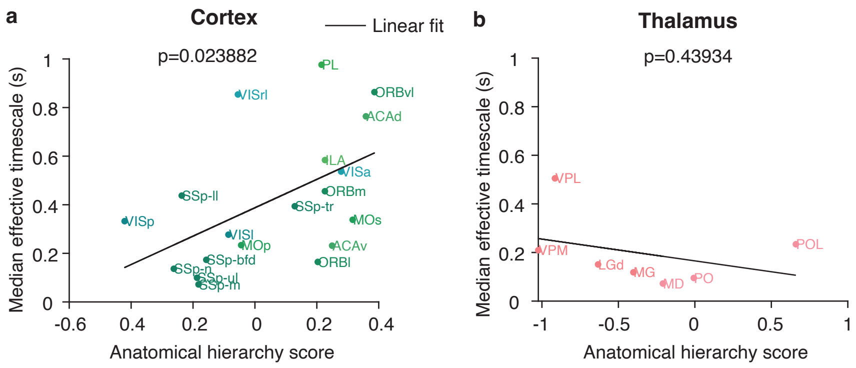

Supplementary Figure 5. Relationship between anatomical hierarchy scores and effective timescales in previously studied regions. **a**, Median effective timescales increased with the anatomical hierarchy scores (Pearson correlation coefficient, $r = 0.52$, $p = 0.02$, $n = 19$) in the subset of cortical areas studied previously [2]. Black line shows the linear fit. **b**, Median effective timescales did not significantly correlate with the anatomical hierarchy scores (Pearson correlation coefficient, $r = -0.35$, $p = 0.44$, $n = 7$) in the subset of thalamic areas studied previously [2]. Black line shows the linear fit.

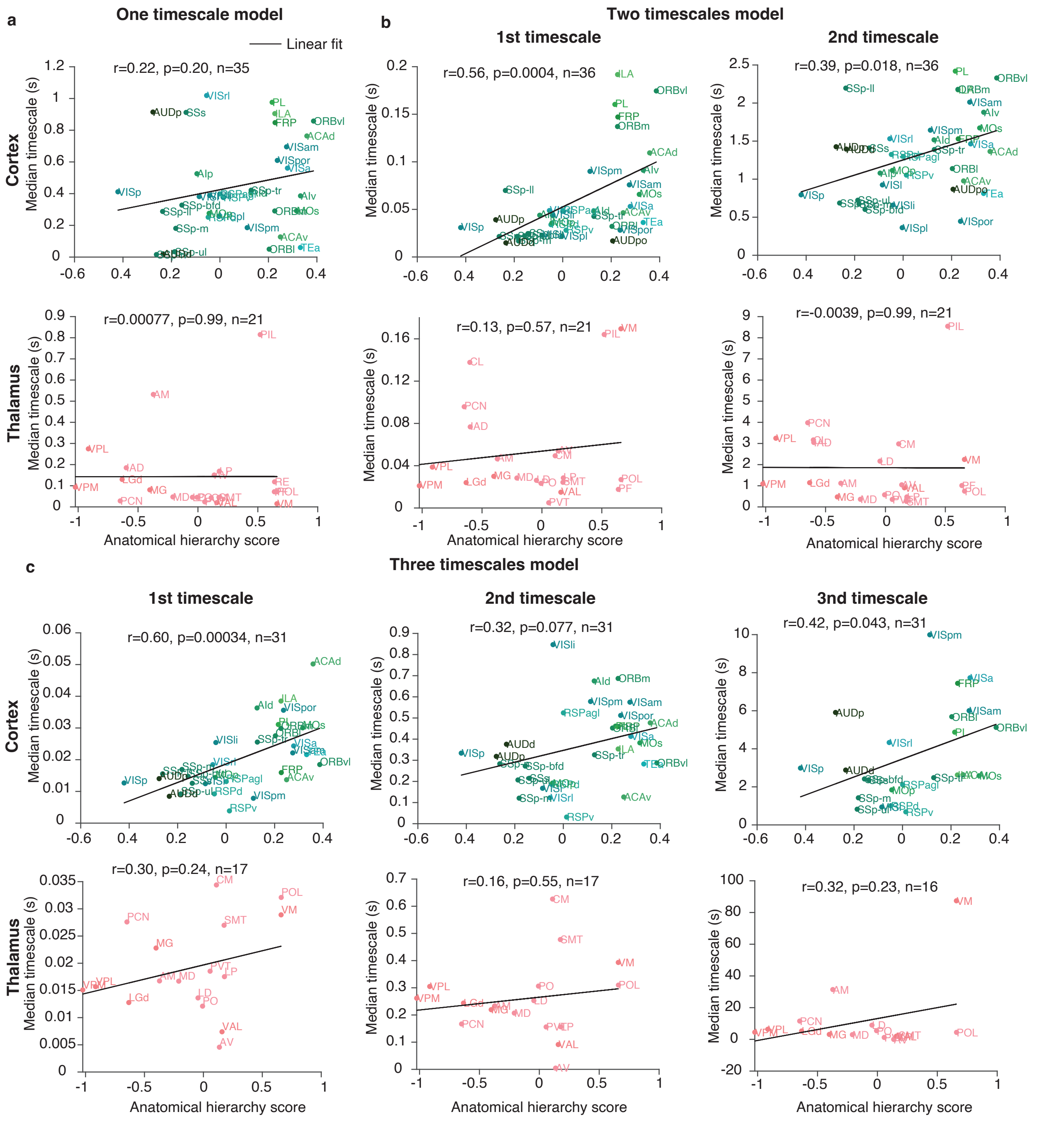

Supplementary Figure 6. Relationship between anatomical hierarchy scores and individual fitted timescales of single neurons. **a**, Relationship between anatomical hierarchy scores and median timescales for single-timescale neurons in cortex (top) and thalamus (lower panel). **b**, Relationship between anatomical hierarchy scores and median constituent timescales (1st and 2nd) for two-timescale neurons in cortex (top) and thalamus (lower panel). **b**, Same as panel b for three-timescale neurons. In all panels, r denotes the Pearson correlation coefficient, p the p-value, and n the sample size. Black lines show the linear fit.

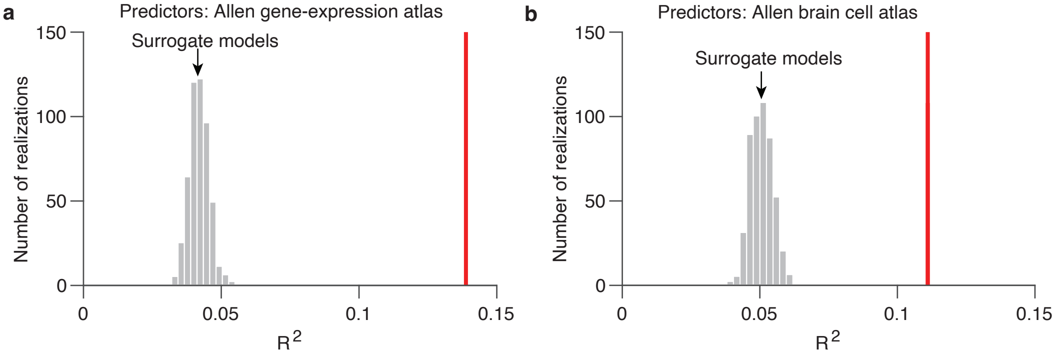

Supplementary Figure 7. Phase-randomization test of models predicting timescales from anatomical features. a, The prediction accuracy R^2 of regression models with predictors from the Allen gene-expression atlas (red line) was significantly higher than that of surrogate models based on phase randomization, which preserves the spatial correlation structure of predictors but eliminates their relationship to the timescale data (gray histogram, 500 simulations). **b**, Same as panel a for predictors from the Allen brain cell atlas.

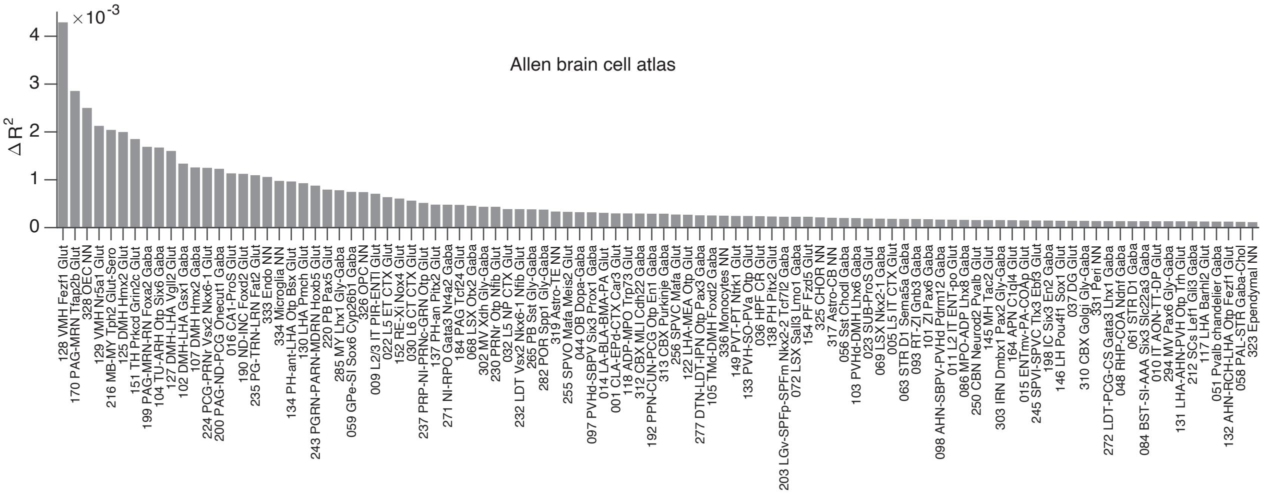

Supplementary Figure 8. Contribution of individual cell types to predicting timescales. Top 100 genetically defined cell types ranked by unique explained variance ΔR^2 in predicting timescales.

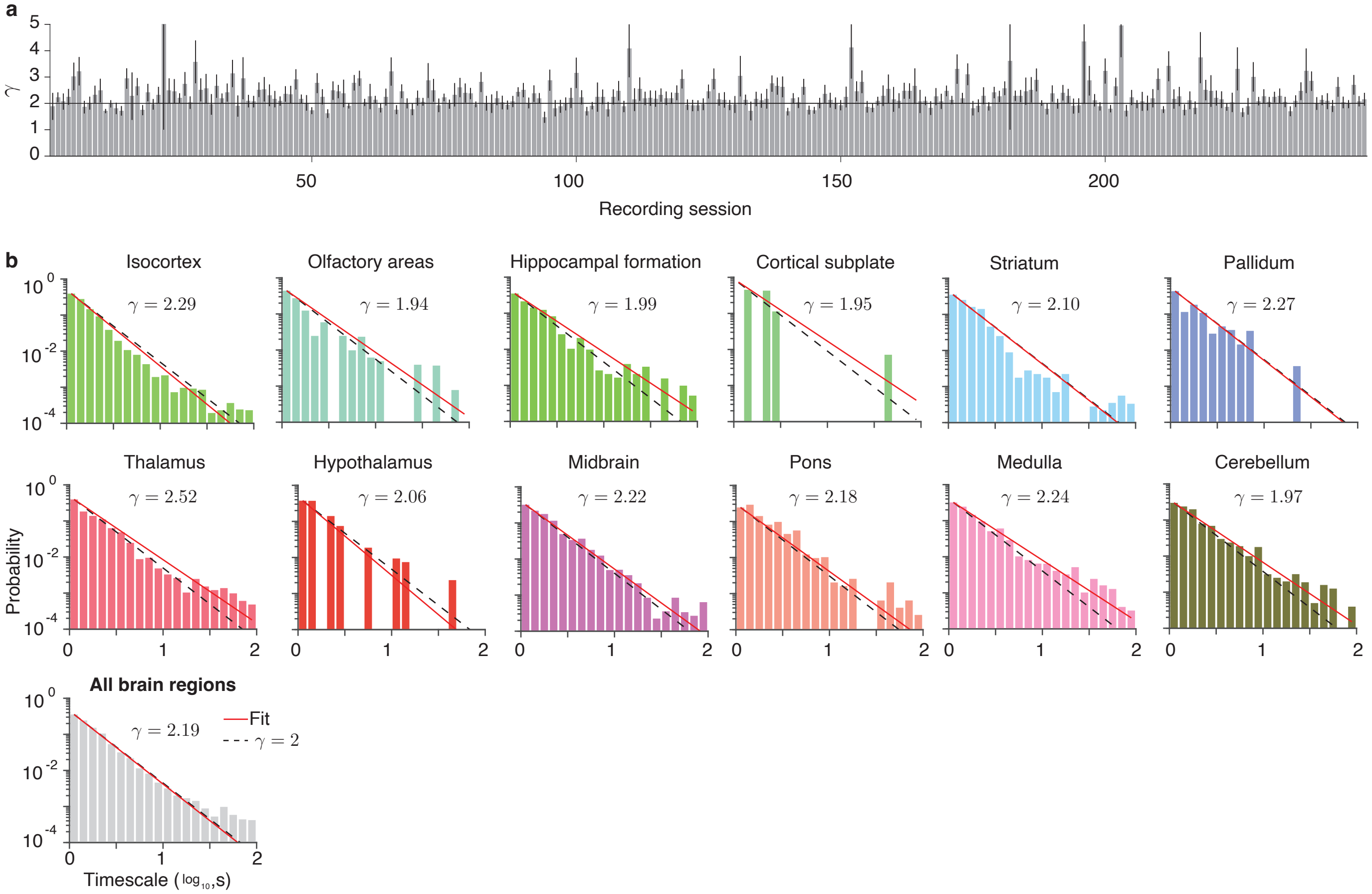

Supplementary Figure 9. Additional tests of the power-law distribution of timescales across the brain. **a**, The exponent γ of the best-fitting power-law for timescale distributions was near 2 in most recording sessions, which had sufficient data for fitting (more than 40 neurons). Error bars represent 95% confidence intervals. The solid horizontal line indicates $\gamma = 2$. **b**, The distributions of timescales estimated from fitting a single-exponential function across neurons within each major brain structure (colored) and across neurons pooled from all regions (gray) are well described by a power law with an exponent γ close to 2 (red line: best-fitting power law; dashed line: reference power law with exponent 2). All neurons were included in this analysis.

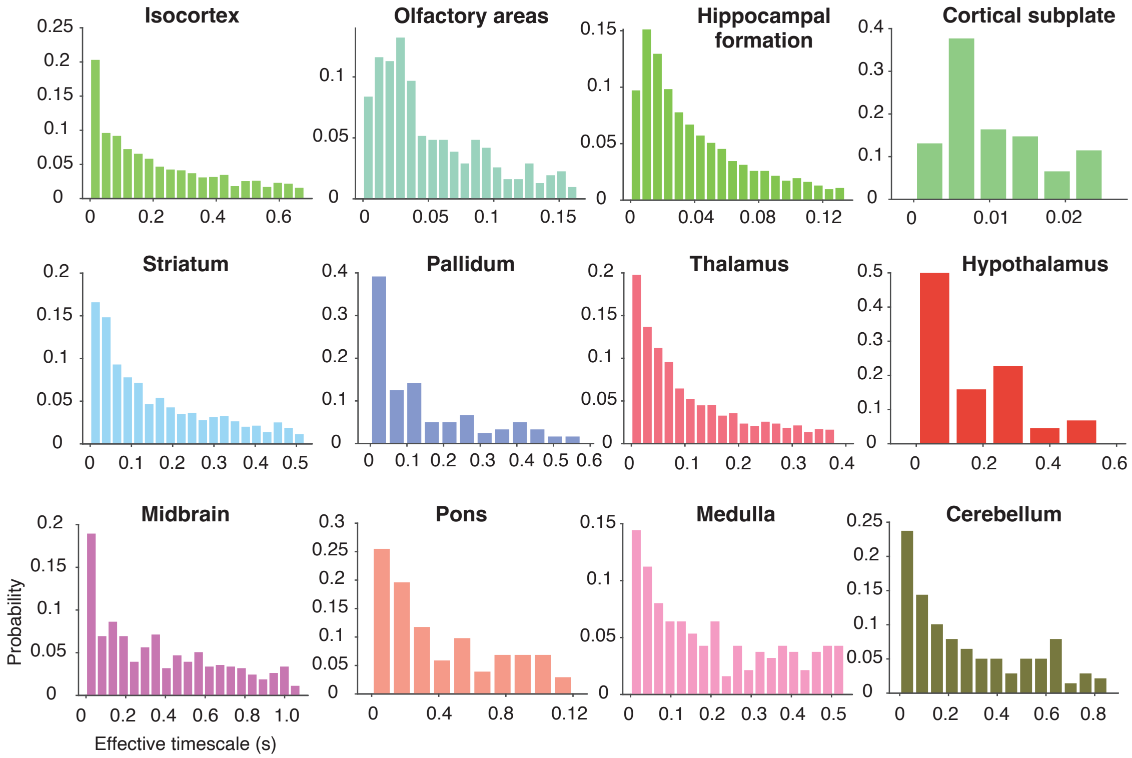

Supplementary Figure 10. Distribution of fast timescales. Distributions of fast timescales below the power-law cutoff, which do not follow a power law, for each of the 12 major brain structures.

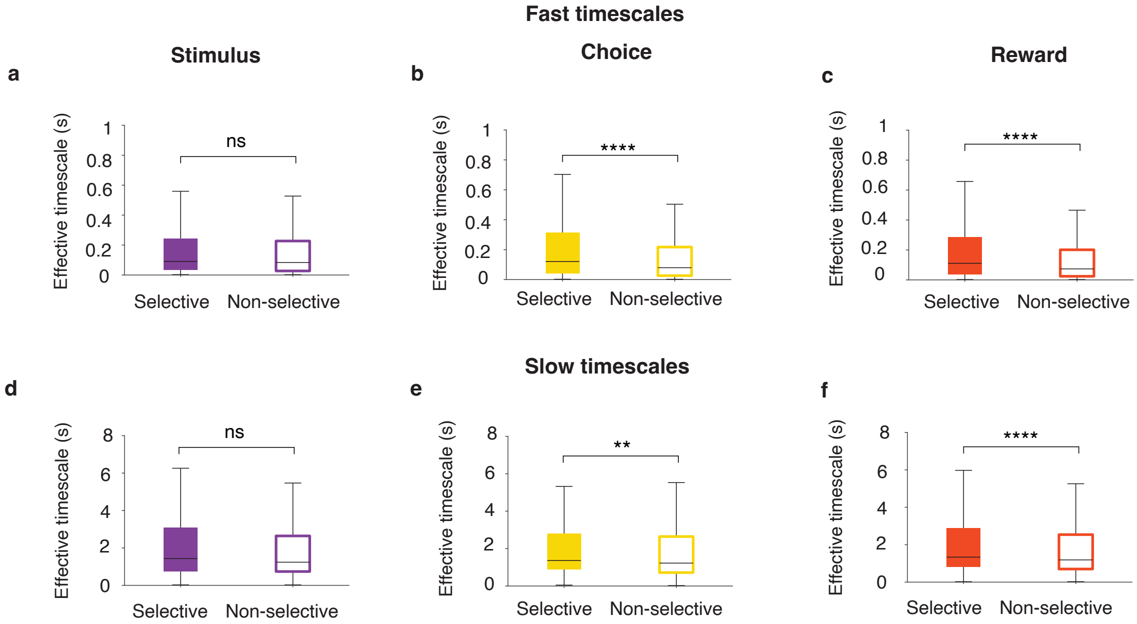

Supplementary Figure 11. Relationships of fast and slow timescales with task-variable encoding. a, Fast effective timescales did not differ significantly between neurons selective (filled bar) and non-selective (open bar) for the visual stimulus ($p = 0.09$, two-sided Wilcoxon rank sum test; $n = 490$ selective, $n = 6,480$ non-selective). In box plots (a-f), center lines indicate medians; boxes span the 25th to 75th percentiles; whiskers extend to the nearest of $1.5\times$ the interquartile range or the most extreme data point. **b**, Fast effective timescales were significantly longer in choice-selective neurons (filled bar) than in neurons non-selective for choice (open bar; $p < 10^{-10}$, two-sided Wilcoxon rank sum test; $n = 839$ selective, n = $6,131$ non-selective). **c**, Fast effective timescales were significantly longer in reward-selective neurons (filled bar) than in neurons non-selective for reward (open bar; $p < 10^{-10}$, two-sided Wilcoxon rank sum test; $n = 2,235$ selective, $n = 4,735$ non-selective). **d**, Slow effective timescales did not differ significantly between neurons selective (filled bar) and non-selective (open bar) for the visual stimulus ($p = 0.10$, two-sided Wilcoxon rank sum test; $n = 268$ selective, $n = 3,389$ non-selective). **e**, Slow effective timescales were significantly longer in choice-selective neurons (filled bar) than in neurons non-selective for choice (open bar; $p = 0.002$, two-sided Wilcoxon rank sum test; $n = 540$ selective, $n = 3,106$ non-selective). **f**, Slow effective timescales were significantly longer in reward-selective neurons (filled bar) than in neurons non-selective for reward (open bar; $p = 7.7 \cdot 10^{-6}$, two-sided Wilcoxon rank sum test; $n = 1,337$ selective, $n = 2,309$ non-selective).

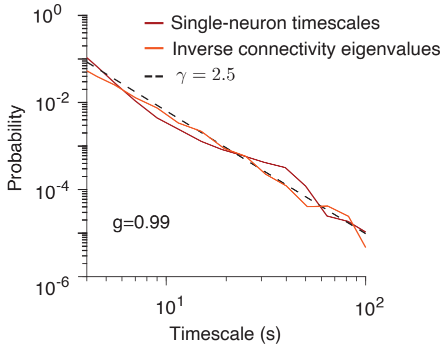

Supplementary Figure 12. Comparison between the distributions of single-neuron timescales and inverse connectivity eigenvalues. In linear networks, the distribution of single-neuron timescales, estimated by fitting their autocorrelation with an exponential function (red), closely matches the distribution of the network timescales given by the inverse eigenvalues of the connectivity matrix (orange). Simulations are shown for a linear network with connection weights sampled from a zero-mean normal distribution, corresponding to a semi-circle distribution of eigenvalues ($\zeta = 0.5$). The synaptic gain parameter is $g = 0.99$. Dashed line: reference power law with $\gamma = 2.5$.

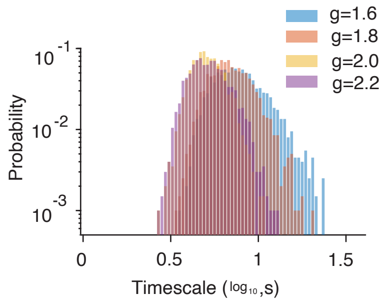

Supplementary Figure 13. Distribution of timescales in nonlinear networks with Gaussian connectivity. Distribution of timescales in nonlinear network models with connectivity weights sampled from a Gaussian distribution. For each value of g, we performed 1,000 simulations, following the procedures for timescale estimation described in the Methods.
